# Lactose tolerance test as an alternative to hydrogen breath test in the study of lactose malabsorption

**DOI:** 10.1515/almed-2020-0102

**Published:** 2020-11-12

**Authors:** Teresa Sendino, Amaia Sandúa, Sofía Calleja, Álvaro González, Estibaliz Alegre

**Affiliations:** Service of Biochemistry, Clínica Universidad de Navarra, Pamplona, Spain; Instituto de Investigación Sanitaria de Navarra (IdiSNa), Pamplona, Spain

**Keywords:** glucose, hydrogen, lactose malabsorption, tolerance test

## Abstract

**Objectives:**

Lactose malabsorption is generally assessed by hydrogen breath testing (HBT). However, this test is not recommended in patients with high baseline hydrogen concentrations (H_2_B). In addition, breath testing is not recommended in the current situation created by the COVID-19 pandemic, due to the potential infectiveness of the samples. The objective is to assess concordance between HBT and lactose tolerance test (LTT) depending on H_2_B concentrations.

**Methods:**

A total of 430 patients (40 years, Q1–Q3 = 28–54 years; 66.7% women) suspected of lactose malabsorption were included in the study. Breath and heparinized blood samples were collected at baseline and sequentially after the intake of 50 g of lactose, to measure hydrogen in breath and glycemia in blood, respectively.

**Results:**

H_2_B was <10 ppm in 69.5% of subjects; 10–20 ppm in 14.7%; and >20 ppm in 15.8% of subjects. In patients with H_2_B <20 ppm, concordance between HBT and LTT was moderate and consistently improved when the cut-off in LTT was set at 15 mg/dL. The increase in hydrogen and glucose correlated negatively (r=−0.389; p<0.05). The increase in glycemia during LTT was not influenced by H_2_B levels obtained in HBT.

**Conclusions:**

LTT emerges as an alternative to HBT to assess lactose malabsorption in the presence of high H_2_B levels or when breath testing is not recommended by the circumstances. The best concordance was obtained when the cut-off for LTT was set at 15 mg/dL.

## Introduction

There is a wide variety of dairy products available in the market, which account for 14% of energy intake in adults in Europe [1]. Lactose, the main sugar of milk, is hydrolyzed in the small bowel by lactase-phlorizin hydrolase into glucose and galactose prior to absorption. Deficient intestinal lactase activity is common in adults, with important variations across ethnic groups. This deficiency is associated with lactase genetic polymorphisms, primarily C/T-13910 polymorphism. Genotypes TT and TC, which are more frequently expressed in the European population, are associated with the preservation of lactase activity [2], [3]. In addition, deficient lactase activity can be secondary to gastrointestinal conditions, such as celiac disease, gastroenteritis and Crohn’s disease [1].

Lactase deficiency causes lactose malabsorption in the small bowel. Unabsorbed lactose is metabolized by colonic bacteria to produce gas such as hydrogen and methane. Manifestations of lactose intolerance after the intake of milk or its derivatives include abdominal pain, bloating, flatulence or diarrhea [4]. About one-third of affected patients develop lactose intolerance [5]. However, as a result of colonic adaptation, most lactose malabsorbers can tolerate some amount of lactose without developing symptoms [6]. Thus, with the intake of small amounts of lactose, the intestinal microbiota adapts through the proliferation of lactase-producing bacteria.

A variety of methods are available to assess lactose malabsorption. The gold-standard method involves measuring lactase activity in a biopsy of the jejunum. However, as this test is highly invasive, clinicians prefer using indirect methods, based on serially measuring glucose in blood (lactose tolerance test, LTT) or expired hydrogen in breath (hydrogen breath test, HBT) after lactose intake [1]. In addition, there are other tests available, such as the genetic test or the gaxylose test [1].

HBT is the most common indirect method for assessing lactose malabsorption. It is the test with the best diagnostic value [7], supported by extensive scientific evidence. Several consensus documents have been recently published that provide guidance on the performance and interpretation of results [8], [9]. An HBT test is positive when breath H_2_ levels increase by 20 ppm with respect to the baseline value in the 3 h following lactose overload [8]. Previous bowel preparation is required, including a fiber- and lactose-free diet in the previous 24 h, and the use of a mouth wash immediately before the test. In addition, the subject cannot take antibiotics or laxatives, use enemas or undergo a colonoscopy in the previous days, as they can alter the intestinal microbiota. In fasting conditions, baseline H_2_ levels (H_2_B) are usually 7±5 ppm [10]. A significant limitation of HBT is the difficult interpretation of results in the presence of an elevated H_2_B (>20 ppm), where this test is not recommended.

In addition, the COVID-19 pandemic caused by SARS-CoV-2 has posed a new limitation to this test. Breath samples are potentially infectious, as they contain viral particles [11] that jeopardize the safety of both, patients and clinicians.

LTT is the most economical test, although it is more invasive [1]. Performance standardization has not yet been established, and a variety of protocols and cut-off values have been proposed to define lactose malabsorption. The most commonly used cut-off point for the increase in glucose concentrations with respect to baseline concentrations is 20 mg/dL [12], although 15 mg/dL has been reported to have better sensibility and specificity [7], [13]. An important limitation is that this test is not valid or indicated for diabetic patients [14], and bacterial overgrowth can interfere with its interpretation.

Previous studies have shown a moderate concordance between HBT and LTT [7], [13], [15]. The objective of this study is to identify the best cut-off values for LTT to improve concordance between the two methods. A secondary objective is to assess the validity of this test as an alternative method when H_2_B is elevated or when this test is not recommended in situations such as in those with a potential risk of SARS-CoV-2 infection.

## Materials and methods

### Patients

The initial sample was composed of 516 patients who had undergone a routine lactose malabsorption study that included HBT and LTT, within a period of three years (March 2007–March 2010). To prevent deviations caused by technical causes, the tests where H_2_ was undetectable in all measurements, were excluded. Tests with baseline glycemia >126 mg/dL were excluded due to potential alterations in glucose metabolism, which could interfere with the interpretation of results. The final sample was composed of 430 subjects (median age 40 years, Q1–Q3=28–54 years; 66.7% women). Patients followed a fiber-, lactose-free diet 24 h before the test and were not allowed to smoke the day of the test. The test was performed with the subjects in fasting conditions. The intake of antimicrobials, laxatives or enemas was not permitted, and subjects could not have a colonoscopy seven days before the test. Immediately before the test, subjects were asked to use a mouthwash. The study was approved by the Institutional Review Board.

### Lactose tolerance test

Subjects ingested 50 g of lactose dissolved in 200 mL of water. This dose does not correspond to the currently recommended dose for HBT, although it was commonly used for LTT in the past. For HBT, breath samples were taken at baseline and at 30 min intervals for 3 h. H_2_ concentration was measured using the Breath Tracker H2+ system (QuinTron, Milwaukee, USA). An increase in hydrogen concentration >20 ppm with respect to baseline was indicative of lactose malabsorption [8]. For LTT, peripheral, heparinized blood samples were drawn at baseline and sequentially at 30 min intervals for 2 h. After centrifugation, plasma glucose levels were measured using the glucose-oxidase method in a modular P system (Roche Diagnostics, Mannheim, Germany). Maximum glycemia increase with respect to baseline was calculated. Three cut-off values of increase with respect to baseline were used to identify lactose malabsorption: 25, 20 and 15 mg/dL.

### Statistical analysis

Data are expressed as median values and interquartile ranges. Non-Gaussian distribution was confirmed by the Kolmogorov-Smirnov test of normality. Comparison across groups was performed by the Kruskal–Wallis test, followed by Dunn’s multiple comparison test. Correlations were assessed by Spearman’s correlation coefficient. Comparison of frequencies was performed by chi-squared test. The level of concordance was assessed by Cohen’s kappa coefficient, considering a value of 0.81–1.00 as “very good”; 0.61–0.80 as “good”; 0.41–0.60 as “moderate” and 0.21–0.40 as “low” [16]. Statistical analyses were performed using IBM SPSS Statistics v20. A two-tail p-value <0.05 was considered statistically significant.

## Results

Of the initial sample of 516 patients, those with baseline glycemia >126 mg/dL and subjects with undetectable H_2_ in breath during the whole test were excluded. Finally, 430 were selected. HBT and LTT results are shown in Table 1. HBT identified lactose malabsorption in 26.7% of patients vs. 57.4% identified by LTT with a cut-off of 25 mg/dL; 43.5% with a cut-off of 20 mg/dL; and 32.3% with a cut-off of 15 mg/dL.

**Table 1:** Concordance of hydrogen breath test (HBT) and lactose tolerance test (LTT) using different cut-off points of glycemia increase with respect to baseline to identify lactose malabsorption. HBT identifies malabsorption in the presence of a H_2_ increase with respect to baseline >20 ppm.

Baseline H_2_ < 10 ppm						
			HBT		Total.	Kappa’s index (95% CI)
			Normal	Malabsorption		
LTT	25 mg/dL	Normal	127	12	139 (46.5%)	0.36 (0.27–0.45)
		Malabsorption	87	73	160 (53.5%)	
	20 mg/dL	Normal	159	19	178 (59.6%)	0.46 (0.36–0.56)
		Malabsorption	55	66	121 (40.4%)	
	15 mg/dL	Normal	180	24	204 (68.2%)	0.54 (0.44–0.64)
		Malabsorption	34	61	95 (31.8%)	
Total			214 (71.2%)	85 (28.8%)	n=299	

Baseline H_2_ between 10 and 20 ppm						
			HBT		Total.	Kappa’s index (95% CI)
			Normal	Malabsorption		
LTT	25 mg/dL	Normal	23	1	24 (38.1%)	0.24 (0.09–0.4)
		Malabsorption	26	13	39 (61.9%)	
	20 mg/dL	Normal	32	2	34 (54.0%)	0.37 (0.16–0.58)
		Malabsorption	17	12	29 (46.0%)	
	15 mg/dL	Normal	40	3	43 (68.3%)	0.52 (0.29–0.75)
		Malabsorption	9	11	20 (31.7%)	
Total			49 (77.8%)	14 (22.2%)	n=63	

Baseline H_2_ < 20 ppm						
					Total.	
LTT	25 mg/dL	Normal			20 (29.4%)	
		Malabsorption			48 (70.6%)	
	20 mg/dL	Normal			31 (45.6%)	
		Malabsorption			37 (54.4%)	
	15 mg/dL	Normal			44 (64.7%)	
		Malabsorption			24 (35.3%)	
Total					n=68	

In relation to baseline H_2_ concentration, a group of 299 (69.5%) subjects exhibited baseline hydrogen levels <10 ppm. Another group of 63 subjects (14.7%) showed baseline levels between 10 and 20 ppm; and a third group of 68 (15.8%) subjects had baseline H_2_>20 ppm.

Of the 362 patients with a baseline H_2_ concentration >20 ppm, 27.3% were identified as lactose malabsorbers by HBT. LTT identified malabsorption in 55% of subjects when the cut-off was set at 25 mg/dL vs. 41.4% when the cut-off was 20 mg/dL, and 31.7% when the cut-off was 15 mg/dL. The concordance analysis between HBT and LTT results yielded a kappa index of 0.33 (95% CI: 0.25–0.41) when the cut-off for glucose was set at 25 mg/dL; 0.44 (95% CI: 0.35–0.53) with a cut-off of 20 mg/dL; and 0.51 (95% CI: 0.42–0.58) with a cut-off of 15 mg/dL. A negative correlation was observed between maximum delta of H_2_ and glucose; therefore, the higher H_2_ increase was, the lower glycemia increase was (r=−0.389; p<0.05).

In the subgroup of subjects with baseline H_2_ <10 ppm, 28.8% were identified as lactose malabsorbers according to HBT. In the case of LTT, the percentage of patients with lactose malabsorption was 53.6% with a cut-off of 25 mg/dL; 40.4% with a cut-off of 20 mg/dL; and 31.8% with a cut-off of 15 mg/dL. Concordance between HBT and LTT yielded a kappa index of 0.36 (95% CI: 0.27–0.45) when the cut-off was 25 mg/dL; and 0.46 (95% CI: 0.36–0.56) when the cut-off was 20 mg/dL; and 0.54 (95% CI: 0.44–0.64) when the cut-off was 15 mg/dL.

In the subgroup of 63 patients with baseline H_2_ concentrations between 10 and 20 ppm, 22.2% showed increases >20 ppm during HBT, which indicated malabsorption. In LTT, 61.9% of subjects were identified to have lactose malabsorption when the cut-off was set at 25 mg/dL; 46.0% with a cut-off of 20 mg/dL; and 31.7% with a cut-off of 15 mg/dL. The analysis of concordance between HBT and LTT yielded a kappa index of 0.24 (95% CI: 0.09–0.4) when the cut-off was set at 25 mg/dL; 0.37 (95% CI: 0.16–0.58) with a cut-off of 20 mg/dL; and 0.52 (95% CI: 0.29–0.75) with a cut-off of 15 mg/dL.

Finally, according to the LTT, of the 68 patients with baseline H_2_ > 20 ppm, 70.6% had malabsorption when the cut-off was set at 25 mg/dL; 54.4% when set at 20 mg/dL; and 35.3% when the cut-off was set at 15 mg/dL.

The percentage of malabsorbers identified by LTT, regardless of the cut-off point used, was always higher in patients with a baseline H_2_ level >20 ppm, as compared to patients with baseline levels of 10–20 ppm. However, this difference was only significant with cut-off values of 25 mg/dL (p<0.01) and 20 mg/dL (p<0.05).

**Figure 1:**
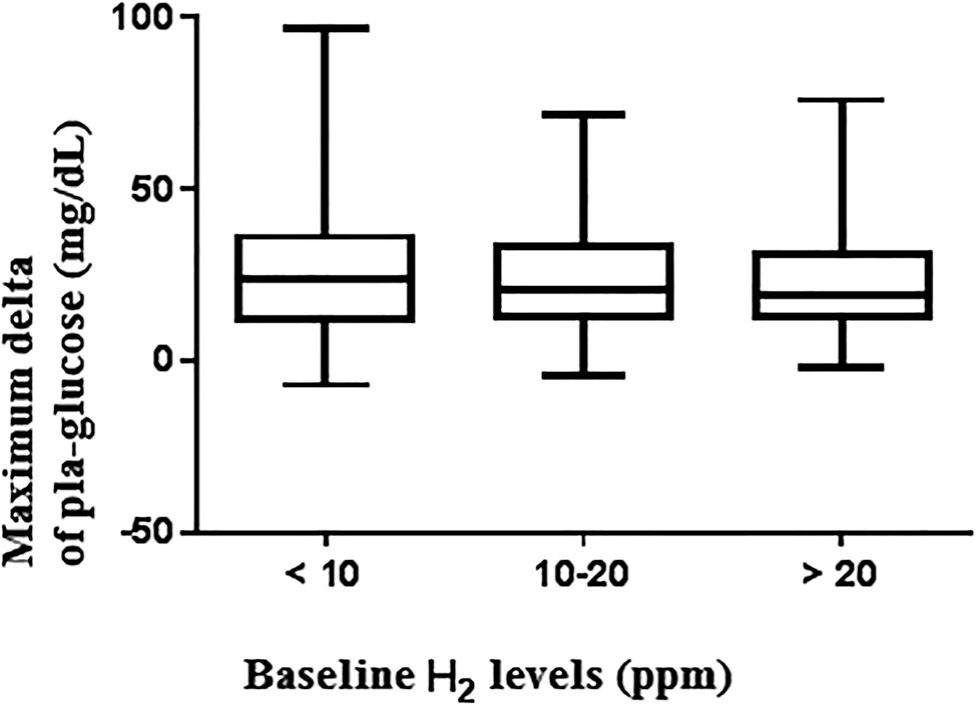
Maximum observed delta of plasma glycemia (mg/dL) during the lactose tolerance test based on baseline H_2_ levels.

The maximum glycemia increase reached during LTT did not vary significantly as a function of baseline H_2_ values (<10 ppm, 10–20 ppm, and >20 ppm) (see Figure 1).

## Discussion

This is one of the largest studies conducted to assess concordance between indirect methods for evaluating lactose malabsorption (HBT and LTT) [7], [15], [17]. Concordance between HBT and LTT was moderate, which may be due to the fact that HBT depends on colonic flora [18], whereas LTT is influenced by physiologic response to glucose. However, concordance between the two tests improved when the LTT cut-off was set at 15 mg/dL in LTT. This cut-off has been proven to have a higher diagnostic efficiency than 20 mg/dL [13].

Having the highest diagnostic efficiency, HBT is the most widely used test, with a mean sensitivity of 77.5% and a specificity of 97.6% [7], [9]. However, this method may yield false negatives due to subject’s inability to produce H_2_ or the intake of antimicrobials; and false positives may also occur due to bacterial overgrowth [1]. Another limitation of HBT is that it is not recommended in the presence of elevated baseline H_2_ values [1]. Some authors establish a cut-off of 10–16 ppm for baseline H_2_ [10]. A relationship has not been observed between the degree of glycemia elevation and baseline H_2_. This may be explained by the fact that glycemia is not influenced by the factors that increase baseline H_2_ production, such as not following the recommended diet or the release of H_2_ retained in the small bowel [19]. As LTT results are independent from baseline H_2_ levels, LTT emerges as an alternative to HBT in patients with elevated baseline H_2_ for whom HBT is not recommended.

LTT can be useful for H_2_-nonproducers, who represent a small proportion of the population [20]. However, this subpopulation of patients can also be identified by simultaneously measuring methane in breath, although this option requires more complex technologies, and a standard measurement method has not yet been established.

In the current pandemic, breath testing is not recommended, as aerosols expelled by COVID-19 patients are an important vector of SARS-CoV-2 transmission, as they contain a high viral load [21]. Obtaining and handling breath samples for HBT involves a high risk. Therefore, it is important that preventive measures for COVID-19 are adopted in the laboratory [22] to protect clinicians and other patients. In contrast, levels of SARS-CoV-2 in blood are low, and sample handling and glycemia tests are highly automated, which reduces the risk of infection substantially. Consequently, this type of samples is considerably safer and reduces the risk of infection.

In this study, the amount of lactose used in the stimulus is higher than the one recommended for HBT [8], although it used to be employed in LTT [1]. The use of 50 g of lactose (approximately the amount in a liter of milk) is higher than the amount generally consumed, and can cause more severe symptoms, especially abdominal discomfort and diarrhea. Unlike HBT, a standard LTT protocol has not yet been established in terms of lactose dose, type of specimen, sampling interval and cut-off value. Some authors have proposed to reduce the duration of the test to 1 h [23] and compare glycemia in capillary blood with glycemia in venous blood, with a modest concordance [24]. A lactose malabsorption test based on 4-galactosylxylose was recently developed, although comparative studies are scarce [25]. On the other hand, the genetic test based on C/T-13910 polymorphism has a higher cost and only identifies subjects with a primary cause of gene underexpression, but not hypolactasia caused by other problems [17]. In addition, no clinical information is provided on patients after exposure to lactose.

In conclusion, this study identifies LTT as an alternative to HBT in the presence of elevated baseline H_2_ or when HBT is not recommended. The best cut-off to guarantee the comparability of results is 15 mg/dL. However, it should be noted that this low cut-off of 15 mg/dL may result in underdiagnosis of lactose malabsorption.
